# Linagliptin Regulates the Mitochondrial Respiratory Reserve to Alter Platelet Activation and Arterial Thrombosis

**DOI:** 10.3389/fphar.2020.585612

**Published:** 2020-11-30

**Authors:** Yi Li, Rong Li, Ziqian Feng, Qin Wan, Jianbo Wu

**Affiliations:** ^1^ Key Laboratory of Medical Electrophysiology of Ministry of Education, Collaborative Innovation Center for Prevention and Treatment of Cardiovascular Disease of Sichuan Province, Drug Discovery Research Center, Southwest Medical University, Luzhou, China; ^2^ Department of Pharmacology, Laboratory for Cardiovascular Pharmacology, School of Pharmacy, Southwest Medical University, Luzhou, China; ^3^ Department of Endocrinology, The Affiliated Hospital of Southwest Medical University, Luzhou, China

**Keywords:** dipeptidyl peptidase-4, linagliptin, cyclic AMP, mitochondria, phosphodiesterases

## Abstract

**Background:** The pharmacological inhibition of dipeptidyl peptidase-4 (DPP-4) potentiates incretin action, and DPP-4 is a drug target for type 2 diabetes and reducing cardiovascular risk. However, little is known about the non-enteroendocrine pathways by which DPP-4 might contribute to ischaemic cardiovascular events.

**Methods:** We tested the hypothesis that inhibition of DPP-4 can inhibit platelet activation and arterial thrombosis by preventing platelet mitochondrial dysfunction and release. The effects of pharmacological DPP-4 inhibition on carotid artery thrombosis, platelet aggregation, and platelet mitochondrial respiration signaling pathways were studied in mice.

**Results:** Platelet-dependent arterial thrombosis was significantly delayed in mice treated with high dose of linagliptin, a potent DPP-4 inhibitor, and fed normal chow diet compared to vehicle-treated mice. Thrombin induced DPP-4 expression and activity, and platelets pretreated with linagliptin exhibited reduced thrombin-induced aggregation. Linagliptin blocked phosphodiesterase activity and contrained cyclic AMP reduction when thrombin stimulates platelets. Linagliptin increases the inhibition of platelet aggregation by nitric oxide. The bioenergetics profile revealed that platelets pretreated with linagliptin exhibited decreased oxygen consumption rates in response to thrombin. In transmission electron microscopy, platelets pretreated with linagliptin showed markedly reversed morphological changes in thrombin-activated platelets, including the secretion of *α*-granules and fewer mitochondria.

**Conclusion: **Collectively, these findings identify distinct roles for DPP-4 in platelet function and arterial thrombosis.

## Introduction

Glucagon-like peptide-1 (GLP-1) is an incretin hormone, and endogenous GLP-1 is rapidly inactivated by dipeptidyl peptidase-4 (DPP-4). The inhibition of DPP-4 with DPP-4 inhibitors is used to improve anti-hyperglycaemic therapy in type 2 diabetes. As a serine protease, DPP-4 cleaves numerous substrates, including fibrin, and DPP-4 can inhibit fibrin polymerization and clot formation (Mentlein and Heymann, 1982). Accumulating evidence showed that DPP-4 inhibition prevented vascular aging, angiogenesis, and atherosclerosis via the modulation of adiponectin/eNOs axis *in vivo* and *in vitro* (Lei et al., 2017; Piao et al., 2017). Increasing evidence indicates the anti-thrombotic effects of GLP-1 receptor agonists and DPP-4 inhibitors. These anti-thrombotic effects were recently confirmed in a model of experimental LPS-induced microvascular thrombus formation in lungs (Steven et al. 2017). Additionally, the loss of DPP-4 activity in endothelial cells upon ischaemia induces a pro-thrombotic status in the endothelium through enhanced tissue factor expression and platelet adhesion (Krijnen et al., 2012).

DPP-4 is an aminopeptidase that has been extensively investigated as a membrane-spanning protein in many tissue cell types and is also found in the plasma in a soluble form. Several potential mechanisms have been proposed to be involved in the thrombosis formation mediated by DPP-4 in the endothelium and in lung injury, including improved endothelial function, decreased free radical production, and reduced inflammation (Krijnen et al., 2012; Beckers et al., 2017). However, the role of DPP-4 in platelet function remains controversial. A recent study revealed that neither MEG-01 cells nor platelets harbor any endogenous DPP-4 activity (Cameron-Vendrig et al., 2016). Interestingly, Gupta et al. (2012) found that sitagliptin, a DPP-4 inhibitor, inhibits platelet aggregation by interfering with intracellular free calcium and tyrosine phosphorylation. Thus, these studies raise the possibility that in activated anucleate platelets, changes in protein expression often do not correlate with changes in corresponding gene transcription (Cimmino et al., 2015). The inhibition of DPP-4 and treatment with a GLP-1 receptor agonist reduce the reactivity of murine and human platelets via a GLP-1 receptor/cyclic AMP (cAMP)/PKA-dependent pathway (Steven et al., 2017). Platelets contain functional mitochondria, and a high level of energy is required in the transition of platelets from a quiescent to an activated state (Holmsen et al., 1982; Verhoeven et al., 1985; Aibibula et al., 2018). Early studies revealed the functional relevance of the interaction between platelet activation and mitochondrial oxidative phosphorylation (OXPHOS). Platelet activation is accompanied by an increased rate of aerobic glycolysis compared with that of OXPHOS, showing that metabolic flexibility changes in platelets.

Linagliptin is a highly potent, competitive inhibitor of DPP-4 in various tissues (Darsalia et al., 2013; Mulvihill and Drucker, 2014; Zhuge et al., 2016). In rodents, linagliptin is more effective than sitagliptin at reducing DPP-4 activity in tissue and related inflammatory properties (Takai et al., 2014; Steven et al., 2015). The importance of platelet homeostasis was emphasized by the recent discovery of platelet mitochondrial dysfunction in type 2 diabetes (Avila et al., 2012; Bhansali et al., 2017). The response of mitochondrial metabolic function to linagliptin remains poorly understood in resting and activated platelets. To address this knowledge gap, we examined whether inhibition of DPP-4 can inhibit platelet activation and arterial thrombosis by preventing platelet mitochondrial dysfunction and release. In the current study, we provide evidence that linagliptin inhibits platelet aggregation in response to thrombin and experimental arterial thrombosis, which was associated with suppressing thrombin-induced cAMP-dependent phosphodiesterase (PDE).

## Materials and Methods

### Animals

C57BL/6J mice were from the Chongqing Medical University Animal Center, Chongqing, China. All protocols for animal use were reviewed and approved by the Animal Care Committee of Southwest Medical University in accordance with Institutional Animal Care and Use Committee guidelines. Five-to six-week-old male C57B6/6J mice were fed a high-fat diet chow (HFC) for 8 weeks, and the mice were injected with solution of *Streptozotocin* (STZ, 55 mg/kg, i.p.). 14 days after STZ injection, the fasting glucose of mice were measured by using an automatic glucometer (Accu-Check; Roche Diagnostics, Mannheim, Germany).

### Carotid Artery Thrombosis Model

Carotid artery thrombosis was produced using ferric chloride (FeCl_3_), as described previously (Ren et al., 2017). Male adult mice were anesthetized by intraperitoneal (IP) injection of sodium pentobarbital (50 mg/kg) and euthanized by rapid cervical dislocation. Linagliptin (ApexBio, United States) prepared in 0.5% hydroxyethylcellulose and was dosed by gavage (5, 10, or 20 mg/kg/day; once daily for 7 days) (Darsalia et al., 2013; Zhuge et al., 2016), and vehicle-treated mice were included. The common carotid arteries were exposed, and filter paper (3 × 1.0 mm) soaked in FeCl_3_ solution (10%) was placed on top of the left carotid artery for 3 min. After the filter paper was removed, the carotid artery was washed in PBS, and the blood flow was continuously monitored with a vascular flow probe (Transonic Systems, Ithaca, NY, United States) from the onset of injury until stable occlusion occurred (defined as no flow for 120 min).

### *In Vitro* Platelet Aggregation

Blood was collected from the inferior vena cava of anesthetized mice into a syringe containing ACD anticoagulant (51 mM tri-sodium citrate, 22 mM citric acid, and 74 mM D-glucose). After centrifugation (100 × g) for 10 min at 22°C, platelet-rich plasma (PRP) was removed, and platelets were pelleted from PRP by centrifugation (400 × g, 10 min, 22°C) and resuspended in 800 µL Modified Tyrode’s-Hepes buffer (134 mM NaCl, 0.34 mM Na_2_HPO_4_, 2.9 mM KCl, 12 mM NaHCO_3_, 20 mM HEPES, 5 mM glucose, 1 mM MgCl_2_, pH 7.3). The platelets in Modified Tyrode’s-Hepes buffer were counted with a haemocytometer, and the concentration was adjusted to 2 × 10^8^ platelets/ml. Platelet aggregation was analyzed with a turbidimetric aggregation-monitoring device (Helena Laboratories, Beaumont, TX, United States). Platelet suspension (300 µl) was incubated with linagliptin (10 or 100 µM) ( Kröller-Schön et al., 2012) or vehicle control (DMSO) for a 40-min pretreatment at 37°C, after which aggregation was induced by the addition of thrombin (0.05 or 0.1 U/ml) under constant stirring (600 rpm). In some experiments, additional pretreatments were performed as detailed (Kirkby et al., 2013). Aggregation to thrombin (0.1 U/ml) was measured after preincubation (1 min, 37°C) with the nitric oxide (NO) donor DEA/NONOate (Sigma) or vehicle.

### Platelet Adhesion Assay

Platelets were labeled with CMFDA (5-chloromethylfluorescein diacetate) (Thermo Fisher Scientific). Platelet suspension (300 µl) was incubated with linagliptin (10 or 100 µM) or vehicle control (DMSO) for a 40-min pretreatment at 37°C, after which adhesion was induced by the addition of thrombin (0.1 U/ml). After that, 20 μl treated platelets solution were dropped to the surface of glass slides. 15 min later, the un-adsorbed platelets were washed away by PBS and the platelets adhered to the surface of glass slides were captured with a fluorescence microscope (Leica). The relative adhesion was measured by pixel density in five microscopic fields in each of three cross-sections of each group using ImagePro Plus software.

### Standard Transmission Electron Microscopy

Washed platelets were fixed by incubating for 1 h at room temperature with 1.25% glutaraldehyde in 0.1 mol/L phosphate buffer at a pH of 7.2, centrifuging for 10 min at 1,100 × g, and washing once in phosphate buffer. Platelets were kept in 0.2% glutaraldehyde at 4°C until processing for standard transmission electron microscopy (TEM) analysis of platelet morphology, as described previously (Adam et al., 2018).

### Mitochondrial Respiration

An Oxygraph-2k (Oroboros, Schroecken, Vorarlberg, Austria) was used to measure the oxygen consumption rate (OCR) of platelets. Washed platelets were suspended in the 2 ml glass chamber at a concentration of 50–300 × 10^6^/ml. Modified Tyrode’s-Hepes buffer was used for experiments in intact cells, while mitochondrial respiration medium MiR05 (110 mM sucrose, 20 mM HEPES, 20 mM taurine, 60 mM K-lactobionate, 3 mM MgCl_2_, 10 mM, KH_2_PO_4_, 0.5 mM EGTA, 1 g/L BSA, pH 7.1) was used for respiration measurements of permeabilized cells (Sjӧ vall et al., 2010; Ehinger et al., 2016). Platelets were stimulated with or without thrombin (0.1 U/ml) in the presence or absence of linagliptin (10 or 100 µM) with a 10-min pretreatment at room temperature.

In experiment for intact cells, respiration was first allowed to stabilize without any additions at a routine state, that is, in the physiological coupling state controlled by cellular energy demands on OXPHOS. Then the ATP synthase inhibitor oligomycin (2.5 µM) was added to reveal respiration independent of ADP phosphorylation (LEAK). To evaluate maximal capacity of the electron transfer system (ETS), the protonophore carbonyl cyanide p-(trifluoromethoxy) phenylhydrazone (FCCP) (0.5 µM each step) was titrated until no further increase in respiration was detected. The ETS was then inhibited by adding rotenone (0.5 µM) (Complex I inhibitor) and antimycin-A (2.5 µM) (Complex III inhibitor), and the residual oxygen consumption was measured.

In experiment of permeabilized platelets, routine respiration was established. Secondly, the detergent digitonin was added to the reaction to change membrane permeability. Mitochondrial respiration (state 2 respiratory rate) was initiated by adding glutamate (5 mM) and malate (5 mM), and OXPHOS through complex I (OXPHOS CI) was started by adding ADP (1 mM). Furthermore, the addition of Cytochrome C was used to evaluate the mitochondrial outer membrane integrity. The simultaneous input of electrons through both CI and complex II (CII) (OXPHOS CI + CII) was achieved by adding succinate (10 mM), which is oxidized by CII. Next, proton leakage over the mitochondrial membrane was measured by the addition of ATP synthase inhibitor oligomycin (2 μg/ml) (LEAK CI + CII). The maximum uncoupled respiration of the electron transport system (ETS CI + CII) was induced by titrating FCCP (1 μM each step). Inhibition of Complex I by rotenone (1 μM) revealed the ETS capacity supported by succinate through Complex II alone (ETS CII). Finally, residual oxygen consumption was determined by addition of a complex III inhibitor antimycin-A (1 μg/ml).The results were analyzed by using DatLab software version 7.3.0.3 (Oroboros Instruments, Innsbruck, Austria). Correction for background and air calibration was performed according to the manufacturer’s instructions.

### Evaluation of Cyclic AMP-Phosphodiesterase Activity and Cyclic AMP Level in Platelets

The washed platelets (2 × 10^8^/ml) were lysed by 0.5% Triton X-100 after stimulation with thrombin or vehicle in the presence or absence of linagliptin (10 or 100 µM). PDE activity, which depends on cAMP, was assessed by a PDE Activity Assay Kit (Colorimetric) (Cat. ab139460, Abcam) according to the manufacturer’s instructions. The values were normalized to total The protein levels assessed with a bicinchoninic acid (BCA) protein assay (Pierce). In some experiments, washed platelets (1.5 × 10^6^/ml) were collected from the samples. The lysates of platelets were dissolved in the cAMP assay buffer and cAMP concentrations were determined by with the use of the cAMP Complete ELISA Kit (Abcam).

### Dipeptidyl Peptidase-4 Activity

DPP-4 activity in the lysates of resting platelets and platelets activated by thrombin was measured by using a DPP-4 activity assay kit (Cat. MAK088, Sigma-Aldrich) according to the manufacturer’s instructions. The values were normalized to the total protein levels assessed with a BCA protein assay (Pierce).

### Western Blotting

Platelets lysates were subjected to SDS-PAGE, and blocked membranes were incubated with anti-DPP4 antibody (R&D Systems). After washing, blots were incubated with horseradish-peroxidase-conjugated goat IgG raised against rabbit IgG (Cell Signaling Technology) and developed with ECL substrate (Pierce). Blots were stripped and re-probed with anti-GAPDH antibody, using similar techniques.

### Statistical Analyses

Data are the mean of triplicate experiments and are presented as the mean ± SEM. The normality of distribution of the two data groups was tested by using the Kolmogorov-Smirnov test (K-S test). Differences between groups were analyzed by Student’s t test (comparisons of two groups) or ANOVA (multiple comparisons) using GraphPad Prism (La Jolla, CA, United States).*p* < 0.05 was considered to represent statistical significance.

## Results

### Linagliptin Inhibits Arterial Thrombosis After Vascular Injury

**FIGURE 1:**
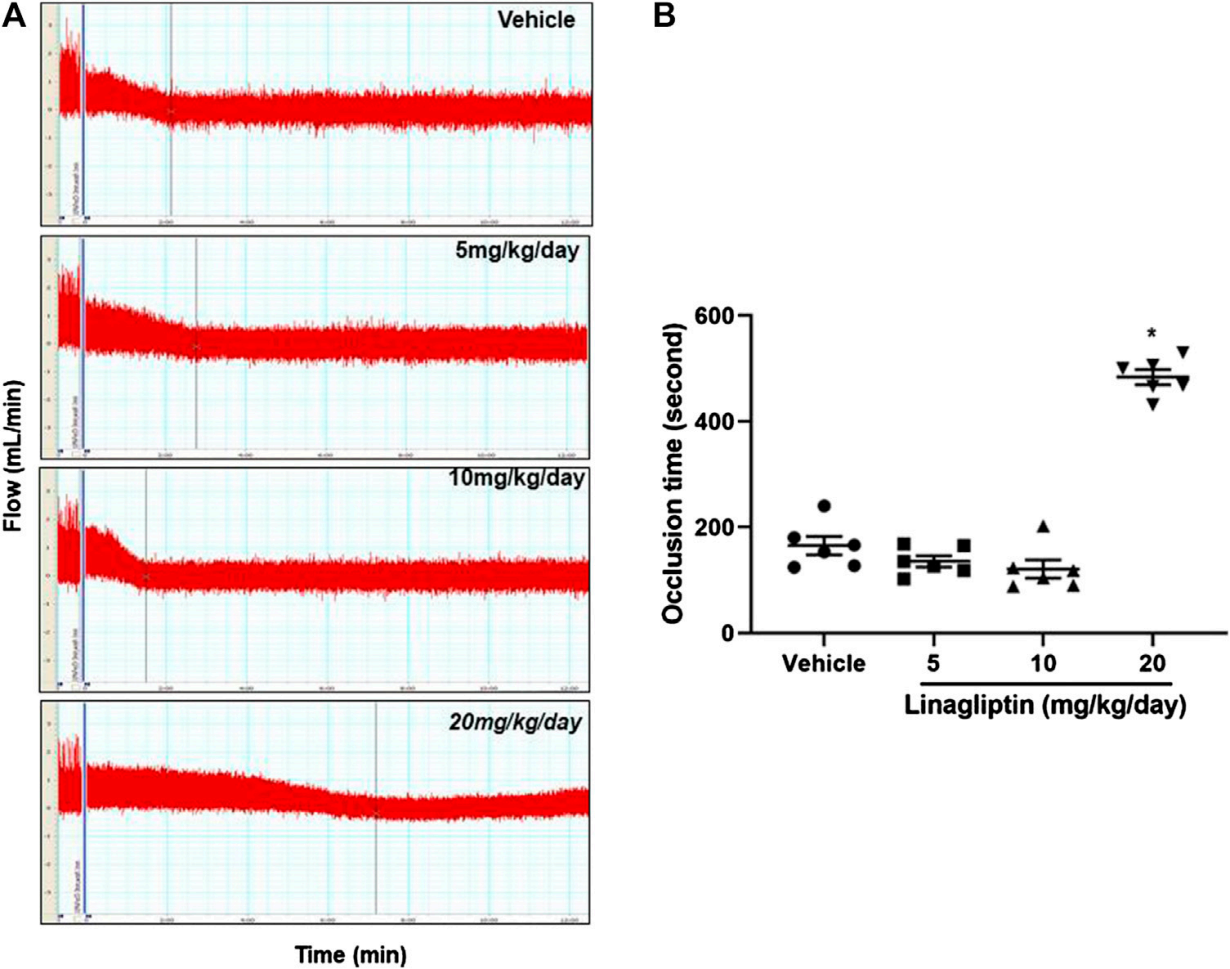
Linagliptin regulates thrombus formation after carotid artery injury in mice fed normal chow. **(A)** Linagliptin or vehicle control was administered by gavage (5, 10, or 20 mg/kg/day; once daily for 7 days) to WT mice before injuring carotid arteries with topical FeCl_3_. Carotid blood flow tracings are shown. **(B)** Mean carotid artery occlusion times after FeCl_3_ injury (n = 6/group); All data were expressed as the means ± SEM. *p < 0.05 vs. 5 and 10 mg/kg/day and vehicle control groups.

Using our well-established common carotid artery injury thrombosis model [18], we determined that compared with the control PBS, linagliptin inhibited thrombus growth in adult male mice. FeCl_3_-induced vascular injury is widely used and rapidly induces the formation of thrombi in an exposed artery. FeCl_3_ diffuses through the vessel wall, resulting in endothelial cell denudation without exposing the inner layers. Mice were divided into four groups; three groups were pretreated with linagliptin (4, 10, or 20 mg/kg/day), and one group was pretreated with vehicle. After 7 days of treatment, thrombus formation was successfully prevented only when the dose of linagliptin was increased to 20 mg/kg/day. The average occlusion time was significantly prolonged in linagliptin-treated mice (479 ± 24.1 s, n = 6) compared with the vehicle mice (163.7 ± 38.2 s, n = 6, *p* < 0.001 vs. linagliptin group) after the initiation of arterial injury (Figures 1A,B). Therefore, linagliptin has an inhibitory effect on the artery thrombosis model, but it is only effective at a high dose (20 mg/kg/day) in mice.

### Linagliptin Inhibits Thrombin-Induced Platelet Adhesion and Aggregation *In Vitro*

**FIGURE 2:**
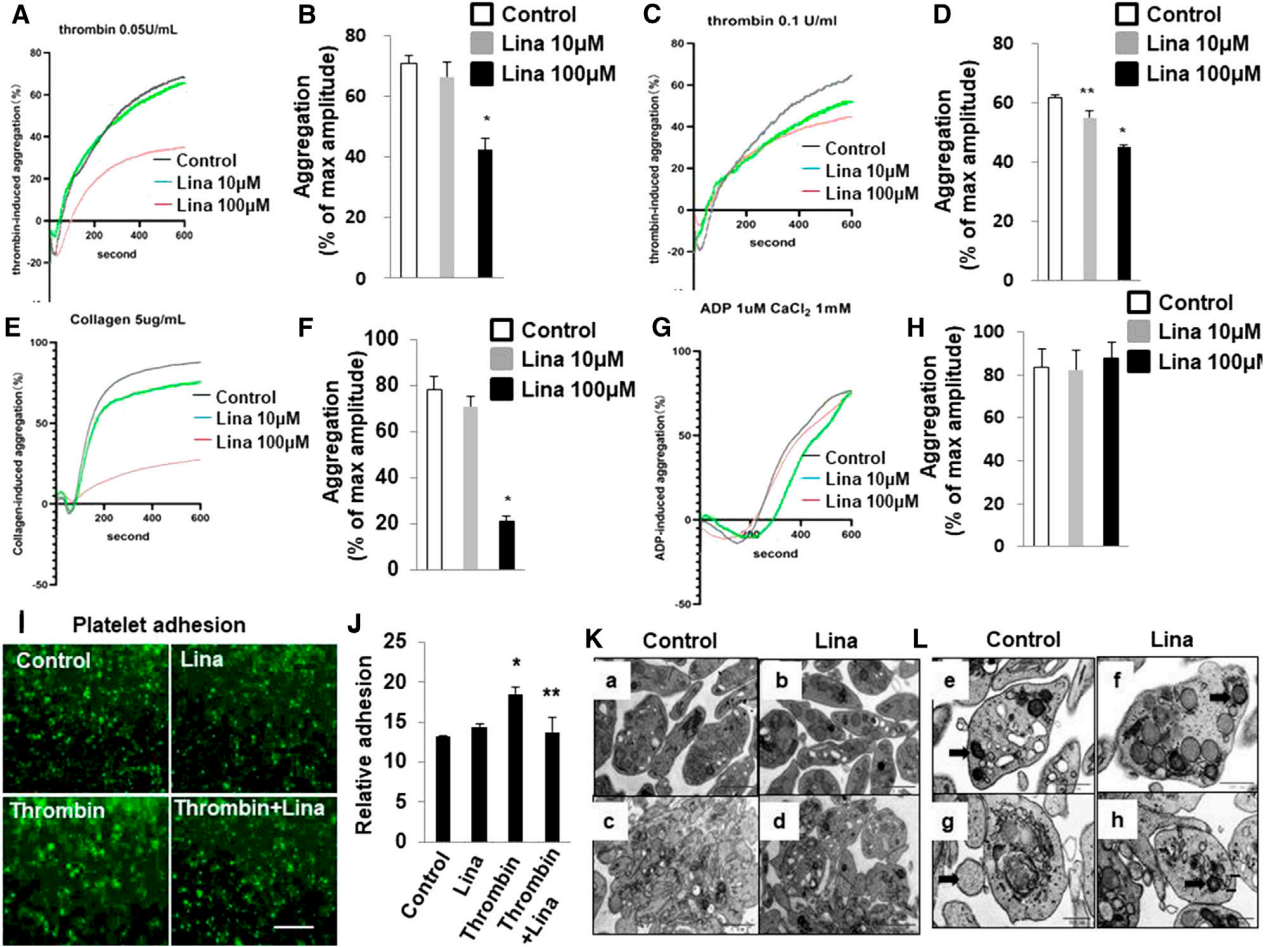
Treatment with linagliptin attenuates thrombin-induced platelet aggregation *in vitro*. Representative aggregometry traces of mouse platelets incubated for 10 min with linagliptin (10–100 μM) and stimulated to aggregate with 0.05 U/ml **(A,B)** or 0.1 U/ml **(C,D)** of thrombin. **(B,D)**: Error bars represent the SEM. n = 4–7/group. *p < 0.05 vs. vehicle and linagliptin (10 μM); **p < 0.05 vs. vehicle. **E–H**: Representative aggregometry traces of mouse platelets incubated for 10 min with linagliptin (10–100 μM) and stimulated to aggregate with 5 μg/ml collagen **(E,F)** and 10 μM ADP **(H,I)**. *p < 0.05 vs. vehicle and linagliptin (10 μM); **(**I,**J)**: Effects of Lina on mice platelet adhesion. **(I)** Representative of images of platelet adhesion after Lina treatment, platelets were pre-labeled with CMFDA, scale bar = 20 μm. **(J)** Quantification of the relative adhesion by pixel density measurements in platelets. All data were expressed as the means ± SEM. Statistical comparisons were performed using the two-way ANOVA. *p < 0.05 vs. vehicle and linagliptin (10 μM). **(K,L)**: Platelets were prepared for transmission electron microscopy imaging, and images were acquired using a Tecnai-12 electron microscope at ×4,300 magnification. **(K)** The distribution of granules (**L**) (scale bar: 1 μm) and mitochondria **(F)** (dark arrow) (scale bar: 500 nm) throughout the platelet. Images are representative of three independent observations. Resting: a,b,e,f; Thrombin: c,d,g,h.

To assess the consequences of DPP-4 inhibition on platelet adhesion and aggregation, mouse platelets were incubated with increasing concentrations of linagliptin (10–100 μM) and stimulated to aggregate with thrombin (0.05 or 0.1 U/ml). Pretreatment with linagliptin inhibited thrombin-induced platelet aggregation. As shown in Figures 2A–D, the results were expressed as % inhibition in a representative aggregation assay. Linagliptin showed dose-dependent inhibition of thrombin (0.1 U/ml)-induced platelet aggregation (Figures 2C,D), whereas linagliptin exhibited only a mild inhibitory effect thrombin (0.05 U/ml)-induced platelet aggregation at a concentration of 10 μM (Figures 2A,B). Similarly, high dose of linagliptin inhibited collagen- but not ADP-induced platelet aggregation (Figures 2E–H). Furthermore, pretreatment with linagliptin (100 μM) inhibited thrombin-induced platelet adhesion (Figures 2I,J). To examine the significance of our findings in a diabetic model, we fed mice high-fat chow (HFD) for 14 weeks, which produced obesity, hyperglycemia. We have addressed the effect of linagliptin on platelet aggregation in diabetic mice. As illustrated in Supplemental Figure S1A,B thrombin (0.1 U)-stimulated platelet aggregation was significantly increased in HFD/STZ mice compared with normal diet (ND) mice (Figures 2C,D) (71.6 ± 5.5% vs. 53.8 ± 2.02%; *p* < 0.05). More importantly, treatment with only high dose of linagliptin (100 µM) significantly inhibited thrombin-stimulated platelet aggregation in platelet samples from HFD mice compared with ND mice. These results demonstrated that platelets from diabetic mice were more sensitive to thrombin-induced aggregation than platelets from non-diabetic mice, and the non-diabetic mice had higher susceptibility to linagliptin-treated aggregation compared to diabetic mice.

The release of granule and respiratory-competent mitochondria induced by various agonists plays key roles in amplifying stimulatory signals and in enabling robust platelet activation at the site of injury (Boudreau et al., 2014; Eckly et al., 2016; Yeung et al., 2018). Thus, we further determined the number of platelet granules by TEM. Resting platelets showed typical morphological characteristics, such as a discoid or round shape and a smooth membrane (Figures 2K,L). In contrast, the number of granules was significantly decreased in platelets stimulated with 0.1 U/ml thrombin compared with resting platelets (4.6 ± 0.46 vs. 9.8 ± 0.85; *p* < 0.01) (Table 1). Pretreatment with linagliptin restored the reduction in granules stimulated by thrombin (10.6 ± 1.04 vs. 4.6 ± 0.46; *p* < 0.05). Similarly, the stimulation of platelets with thrombin resulted in dramatic morphological changes and increased the release of mitochondria, and treatment with linagliptin inhibited the release of mitochondria (Table 1). Thus, the inhibition of DPP-4 inhibits platelet activation and influences platelet components, including granules and mitochondria.

**TABLE 1:** The number of granules and mitochondria in resting and activated platelets.

No. of platelets	Granules/per platelet		Mitochondria/per platelet	
	No	*p*	No	*p*
Resting platelets (11)	9.8 ± 0.85	—	3.5 ± 0.22	—
Thrombin (13)	4.6 ± 0.46	< 0.01	1.3 ± 0.28	< 0.05
Thrombin + Linagliptin (8)	10.6 ± 1.04	< 0.05	3.4 ± 0.37	< 0.05
Linagliptin (9)	9.6 ± 0.78	< 0.05	2.8 ± 0.37	< 0.05

Values are presented with SEM. *p* values were determined by using the two-way analysis of variance to compare each number of granules and mitochondria to resting and thrombin-activated platelets.

### Effect of Linagliptin on Mitochondrial Respiration in Intact Mouse Platelets

**FIGURE 3:**
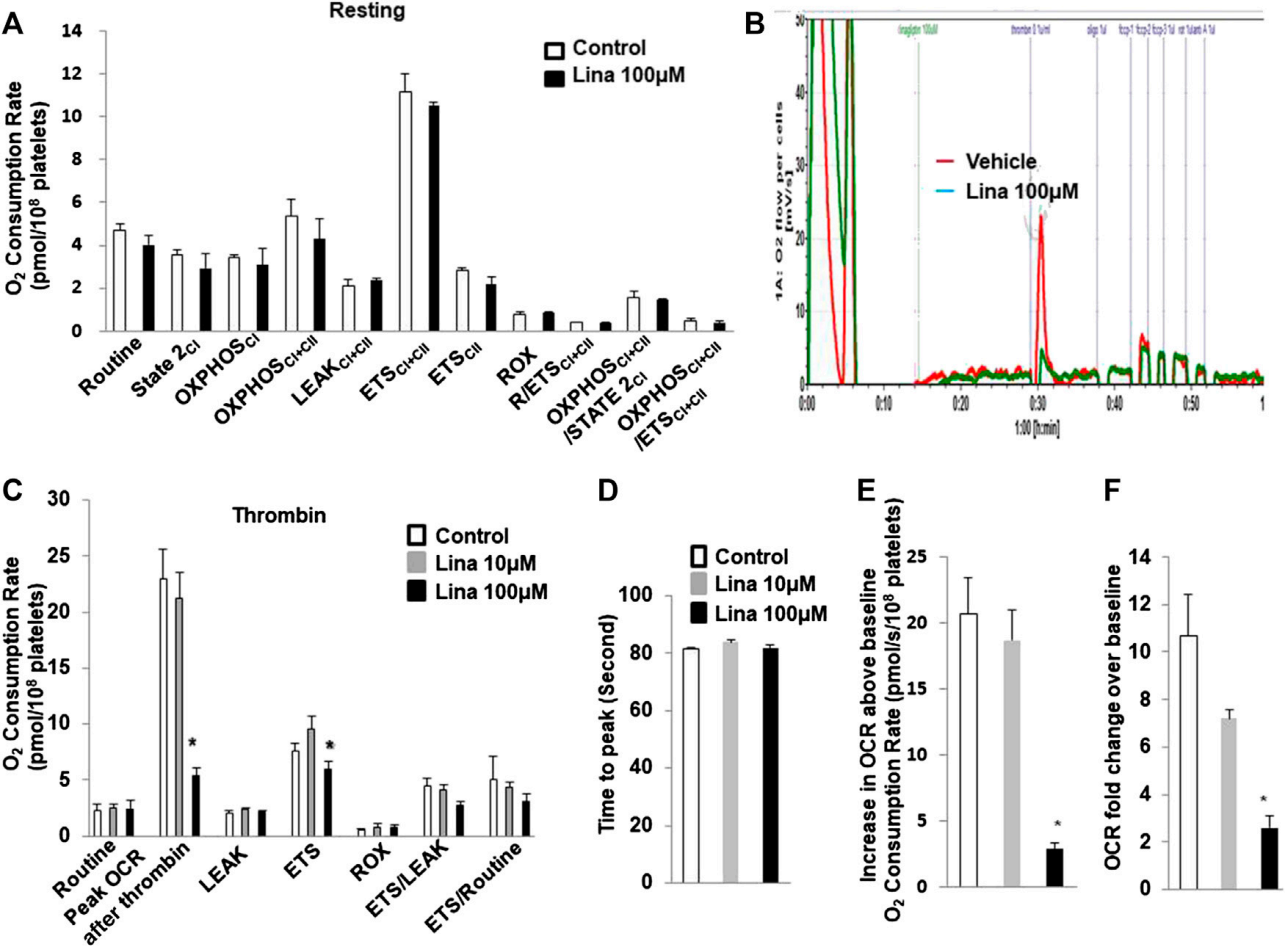
Thrombin promotes an increase in oxygen consumption rate (OCR), which is inhibited by linagliptin. Washed platelets were pretreated with linagliptin (100 μM) or vehicle (DMSO, 0.1%; “control”) for 10 min and then stimulated with thrombin (0.1 U/ml) for 5 min in an Oxygraph-2k high resolution respirometer. **(A)**. Resting platelets were assayed for oxygen consumption after linagliptin treatment; **(B)** Representative trace showing the typical pattern of OCR and depicting the basal level, proton leak, maximal respiration and reserve capacity after pretreatment with linagliptin (100 μM) for 10 min and stimulation with thrombin (0.1 U/ml) for 5 min **(C)**. Platelets were assayed for oxygen consumption under thrombin-stimulated conditions. Time to peak **(D)**, increase in OCR baseline **(E)**, and OCR fold change over baseline **(F)** are shown. Data are presented as the mean ± SEM. n = 3/each group. Statistical analysis: one-way ANOVA with Bonferroni’s multiple comparisons test. *p < 0.05 vs. vehicle. CI, Complex I-linked substrates; CI + II, Complex I + II-linked substrates; ETS, maximal electron transfer system capacity; ROX, residual oxygen concentration; LEAK, leak-state respiration (non-ADP-stimulated respiration); OXPHOS, oxidative phosphorylation capacity (ADP-stimulated respiration).

To determine whether the inhibitory effect of linagliptin on platelet aggregation is associated with a change in mitochondrial metabolism, the OCR, which is an indirect measure of mitochondrial OXPHOS, was calculated with an Oxygraph-2k analyser. Routine respiration was established. Then, washed mouse platelets were stimulated with or without thrombin (0.5 U/ml) in the presence or absence of linagliptin (10 or 100 µM) with a 10-min pretreatment. We first investigated the effects of linagliptin on mitochondrial metabolism in resting platelets. Both basal and peak OCR were unchanged following acute treatment (Figure 3A). Prior to stimulation, the baseline OCR was 2.33 ± 0.53 pmol/10^8^ platelets. Thrombin induced a rapid increase in OCR, and the peak was 23 ± 2.59 pmol/10^8^ platelets higher than the pre-stimulation baseline (Figures 3B,C). The peak increase in OCR was significantly inhibited by linagliptin at a dose of 100 μM (5.35 ± 0.7 pmol/10^8^ platelets). Proton leak was not changed in response to thrombin in the presence or absence of linagliptin. Furthermore, the increase in OCR above baseline was significantly reduced by treatment with linagliptin (100 μM) compared with the control condition and linagliptin treatment (10 μM) (Figures 3E,F). However, the time to peak remained unaltered by any of the tested doses (Figure 3D).

### Effect of Linagliptin on *In Vivo* Mitochondrial Respiration in Treated Mouse Platelets

**FIGURE 4:**
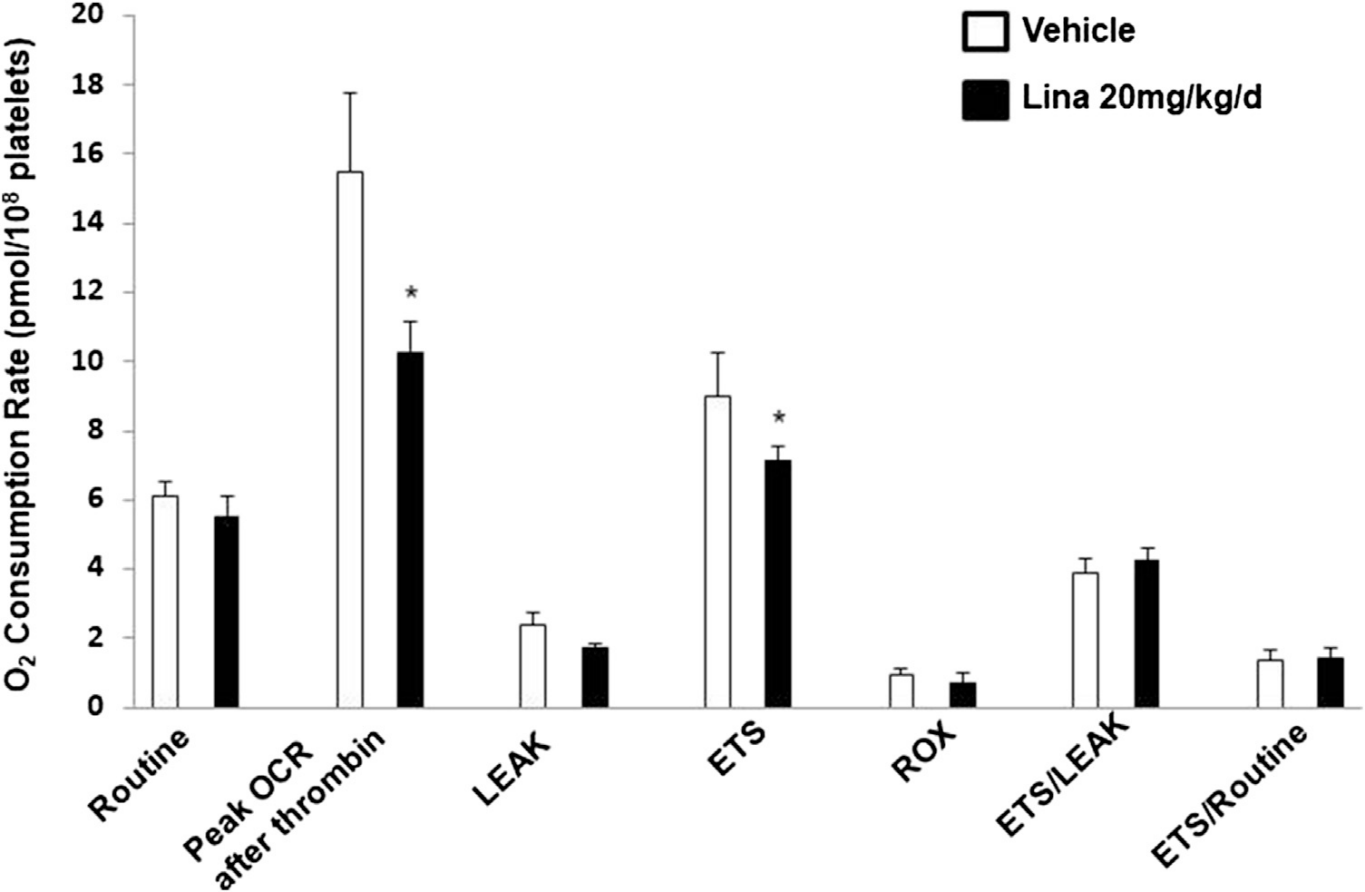
Effect of linagliptin on *in vivo* mitochondrial respiration in treated mouse platelets. Platelets were obtained from mice after 7 days of treatment with linagliptin or vehicle. Platelets were assayed for oxygen consumption in an Oxygraph-2k high resolution respirometer. All data were expressed as the means ± SEM. n = 3/each group. Statistical comparisons were performed using the paired Student’s t-test. Data from graph are normally distributed according to Kolmogorov-Smirnov normality test. *p < 0.05 vs. vehicle. ETS, maximal electron transfer system capacity; ROX, residual oxygen concentration; LEAK, leak-state respiration (non-ADP-stimulated respiration); OXPHOS, oxidative phosphorylation capacity (ADP-stimulated respiration).

To further verify the observation of inhibited thrombus formation following treatment with linagliptin *in vivo*, mitochondrial respiration in intact blood platelets was measured in mice after 7 days of treatment with linagliptin. Platelets from mice treated with linagliptin exhibited similar mitochondrial changes to the *in vitro* findings, characterized by reduced OCR during activation (Figure 4). For comparison, no significant change was observed in mitochondrial function, including oligomycin, FCCP and rotenone/antimycin A (CI/III). These findings support the hypothesis that linagliptin-treated platelets have a decreased effect on mitochondrial respiration during periods of metabolic stress induced by potent platelet agonists.

### Linagliptin Preserves the Activation of Cyclic AMP-Phosphodiesterases Induced by Thrombin

**FIGURE 5:**
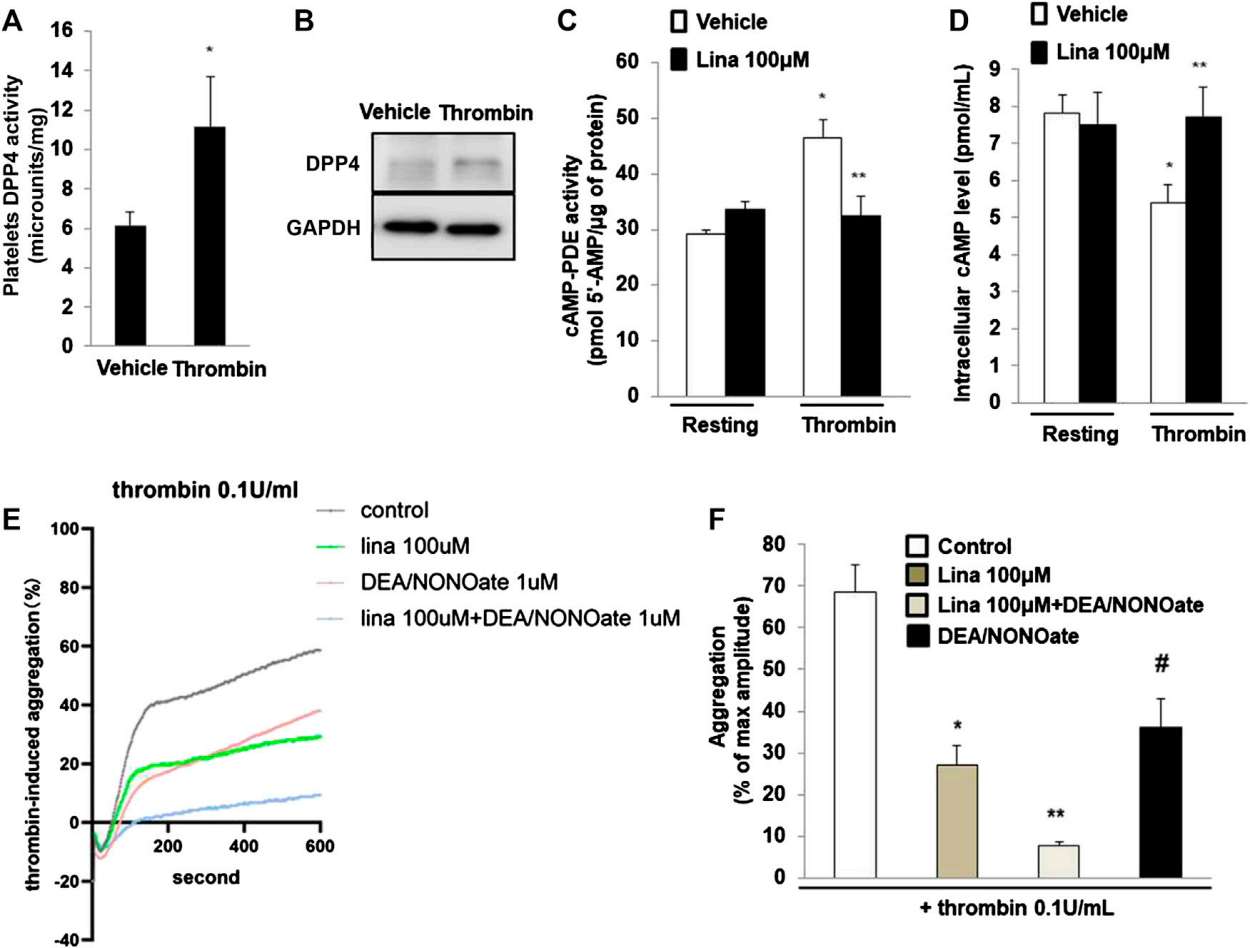
Linagliptin preserves the activation of cyclic AMP (cAMP)-phosphodiesterases (PDEs) induced by thrombin. **(A)**. dipeptidyl peptidase-4 (DPP-4) activity was measured in lysates of platelets treated with thrombin (0.1 U/ml for 10 min) or vehicle. Data are the mean of triplicate experiments and are presented as the mean ± SEM. Data from graph are normally distributed according to Kolmogorov-Smirnov normality test. **(B)**. Expression of DPP-4 in in lysates of platelets was evaluated by western blot analyses. GAPDH was used as an internal control. Image is representative of three independent experiments. PDE activity **(C)** and intracellular cAMP level **(D)** measured in lysates of platelets treated with thrombin (0.1 U/ml for 10 min) in the presence or absence of linagliptin (100 μM). Data are presented as the mean ± SEM. Statistical analysis: one-way ANOVA with Bonferroni’s multiple comparisons test. *p < 0.05 vs. vehicle; **p < 0.05 vs. thrombin alone. n = 3–6/group. **(E**,**F)**. Linagliptin increases the inhibition of platelet aggregation by nitric oxide (NO). Mouse platelets were pretreated with 1 mM DEA-NONOate for 15 min. Representative aggregometry traces of platelets incubated for 10 min with linagliptin (100 μM) and stimulated to aggregate with 0.1 U/ml of thrombin. All data were expressed as the means ± SEM. n = 3–4/group. All data were expressed as the means ± SEM. Statistical comparisons were performed using ANOVA. *p < 0.05 vs. thrombin alone; **p < 0.05 vs. linagliptin; #*p* < 0.05 vs. thrombin alone and linagliptin.

Previously, Cameron-Vendrig et al. reported that DPP-4 activity was not detectable in platelets, but was detected in human plasma [5]. We determined DPP-4 activity following stimulation with thrombin (0.1 U/ml for 10 min). The results showed that DPP-4 activity in the lysates of platelets treated with thrombin was significantly higher than that in the lysates of the control group (Figure 5A). Consistent with this finding, thrombin or vehicle control was added for 10 min, after which platelet lysates were prepared, and assessed by Westernblot analysis. Thrombin stimulated a marked increase in DPP-4 expression in platelets (Figure 5B), suggesting an important role for DPP-4 in the activation of platelets.

PDEs are major targets of antiplatelet therapy to regulate platelet function through the hydrolysis of endogenous cAMP or cGMP (Rosenstock et al., 2019). We examined PDE activity in platelet lysates by using a cAMP-PDE enzymatic activity assay. The results showed that thrombin induced PDE activity, while pretreatment with linagliptin preserved PDE activity (Figure 5C). We further examined the intracellular cAMP level in platelets. Compared with resting platelets, we found that platelet intracellular cAMP level is decreased when thrombin stimulates platelets. The decrease in intracellular cAMP content was significantly blocked when the platelets were pretreated with linagliptin (100 μM). We added the result in revised Figure 5D. In summary, treatment with linagliptin produced cAMP and stimulated PDE, which degraded cAMP. These results suggested that linagliptin prevented cAMP accumulation by preserving PDE activity in platelets.

Previous studies have shown that platelets release NO to regulate aggregation responses (Freedman et al., 1999; Kirkby et al., 2013). To determine whether NO signaling is implicated in DPP-4 inhibition for thrombin-induced platelet aggregation, we performed an additional experiment involving NO signaling in platelet aggregation. The NO donor DEA/NONOate was used to spontaneously releaes NO on platelet aggregation. As shown in Figures 5E,F, thrombin (0.1 U/ml) significantly promoted platelet aggregation. Pretreatment with linagliptin or DEA/NONOate exhibited a similar inhibitory effect on thrombin-induced platelet aggregation. Importantly, addition of both linagliptin and DEA/NONOate showed strong inhibition of thrombin-induced platelet aggregation. These results showed the interaction between NO and DPP-4 inhibition.

## Discussion

DPP-4 is a widely expressed cell surface peptidase that exhibits complex biological functions, including the cell membrane–associated activation of intracellular signal transduction pathways. Linagliptin is a DPP-4 inhibitor used for the treatment of type 2 diabetes mellitus. Similar to other DPP-4 inhibitors, such as sitagliptin and saxagliptin, linagliptin delays the breakdown of endogenous incretin hormones such as GLP-1 and glucose-dependent insulinotropic polypeptide. Linagliptin is effective for glycaemic management in type 2 diabetes, but its cardiovascular safety has not been established (Rosenstock et al., 2019). Little is known about the effect of linagliptin on platelet functions. In this study, we demonstrated a critical role for linagliptin in the regulation of platelet activation and artery thrombosis, suggesting its potential pharmacological use in the prevention and treatment of cardiovascular diseases. Thrombin is a potent platelet activator that may stimulate the activation of platelets and the secretion of platelet granular contents and that may induce aggregation. Linagliptin inhibited the platelet aggregation, mitochondrial respiration function, and PDE induced by thrombin. This finding suggests that mitochondrial respiration is crucial for thrombin-induced platelet aggregation and that linagliptin attenuated platelet aggregation by inhibiting mitochondrial respiration.

**FIGURE 6:**
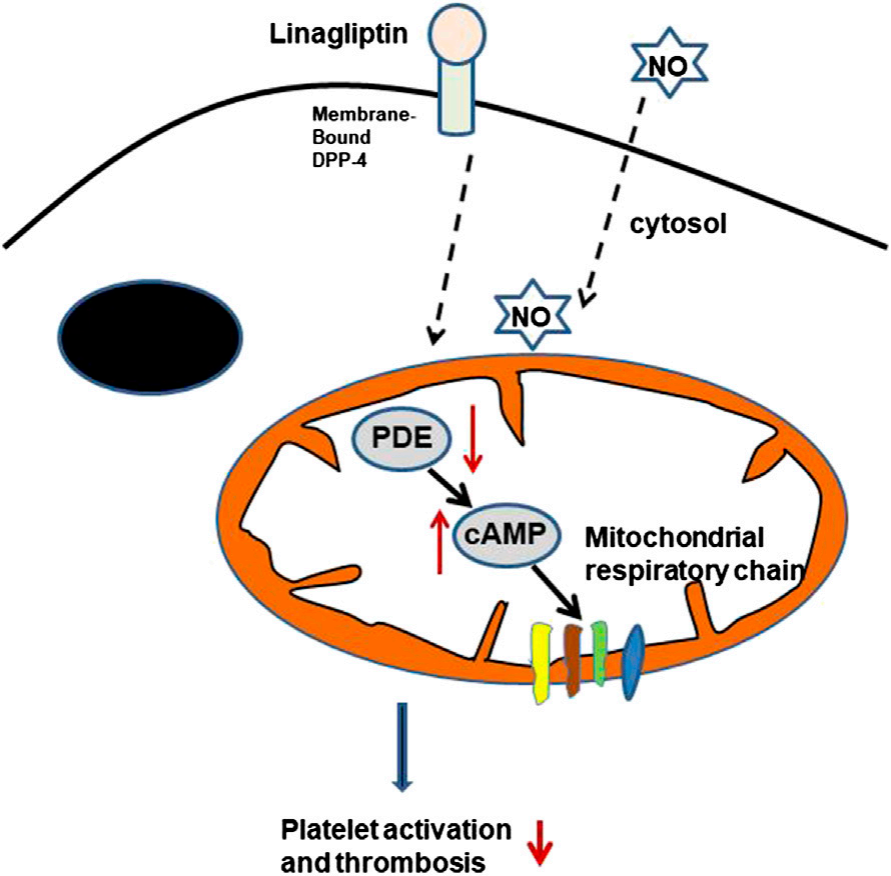
Potential GLP-1-independent mechanism of DPP-4 inhibition by linagliptin in the regulation of platelet activation. cAMP is formed inside the mitochondrial matrix and stimulates the respiratory chain. The cAMP signals are terminated through degradation by PDE in the presence of linagliptin. cAMP, cyclic AMP; PDEs, phosphodiesterases; NO, nitric oxide.

DPP-4 plays an important role in the homeostasis of the gut, and three cell types are considered the main sites of DPP-4 expression and activity: enterocytes, cells of haematopoietic origin, and endothelial cells (Hansen et al., 1999; Mulvihill and Drucker, 2014; Mulvihill et al., 2017). However, little is known about the effect of linagliptin on platelet activity. DPP-4, a serine protease, exists in two forms, a membrane-bound form and a circulating soluble form. Membrane-bound DPP-4 initiates intracellular signaling through interactions with many binding partners and extracellular matrix components. The inhibition of DPP-4 could lead to the pleiotropic actions of DPP-4, such as anti-inflammatory, anti-fibrotic and antioxidant effects (Panchapakesan and Pollock, 2015; Uchii et al., 2016). We report evidence that linagliptin suppresses thrombin-induced platelet activation, which is associated with a reduction in markers of arterial thrombosis formation independent of glycaemic control. Most of the platelet cAMP production induced by agonists was associated with the regulation of mitochondrial function and aggregation. Linagliptin reduced thrombin-induced PDE activation, thus enhancing cAMP-mediated signaling. The significance of cAMP-mediated signaling in the platelet activation induced by linagliptin in our study may partly explain why linagliptin may suppress thrombin-induced mitochondrial cAMP-dependent PDE responses and therefore attenuate platelet aggregation. Alternatively, the interaction between DPP-4 blockade and NO was related to platelet activation (Figure 6).

Mitochondria provide the necessary energy for platelet reactivity and thrombosis-related events (Jobe et al., 2008; Liu et al., 2013). The release of granules facilitates the aggregation of platelets. Stimulated platelets release respiratory-competent mitochondria into the extracellular milieu (Boudreau et al., 2014) and are associated with ATP production. The decreased mitochondrial count in activated platelets compared with resting platelets happened when mitochondria were released or consumed (Joseph et al., 1989; Boudreau et al., 2014; Eckly et al., 2016). To our knowledge, there are no data from animal studies indicating that DPP-4 inhibition has an effect on the regulation of the mitochondrial respiratory chain in platelets. We observed that linagliptin treatment restored the release of granules and the decrease in mitochondrial numbers activated by thrombin, which may result from the pro-thrombotic state of diseases. The current study demonstrates for the first time the involvement of DPP-4 inhibition in that regulation of mitochondrial respiratory capacity and suggests an important biological function in the context of platelet activation and thrombosis. Further studies are necessary to determine the mechanisms by which DPP-4 inhibition regulates the association between mitochondrial function and platelet aggregation.

GLP-1 binds to G-protein-coupled receptors on the cells, leading to increased intracellular cAMP levels and the activation of protein kinases. Since numerous studies have shown that the inhibition of DPP-4 elevates GLP-1/GIP levels, this approach is widely used to treat type 2 diabetes (Mulvihill et al., 2017). DPP-4 inhibition induced by sitagliptin improved renal blood flow and endothelium-dependent relaxation in renal arteries by activating cAMP or GLP-1R signaling (Liu et al., 2012). Further study will evaluate the level of plasma GLP-1 and GLP-1 receptor expressions of platelet in our model in future.

In the present study, we observed that 1 week of linagliptin treatment reduced the platelet mitochondrial respiration induced by thrombin *in vitro* and improved the carotid artery thrombosis model *in vivo*. The preservation of PDE activity in platelets after linagliptin treatment might contribute to the decreased platelet activation. Furthermore, AKT was reported to directly activate PDE (Han et al., 2006; Zhang and Colman, 2007). Treatment of mice fed high-fat chow with linagliptin restored the degree of AKT phosphorylation in cardiac tissues (Al Zoubi et al., 2018). Therefore, it is possible that linagliptin regulates thrombin-mediated AKT in platelets through the activation of cAMP-PDE signaling, indicating that the attenuation of thrombin-induced platelet aggregation induced by linagliptin is possibly correlated with the activation of cAMP production. We also found that linagliptin effectively inhibited the platelet aggregation induced by 0.1 U/ml thrombin but not 0.05 U/ml thrombin, suggesting differential inhibitory effects at varying thrombin levels. The observation that linagliptin suppressed the aggregation induced by collagen or thrombin, but had no effect on similar platelet responses induced by ADP, suggested that DPP-4 inhibition does not directly contribute to fibrinogen receptor activation by ADP. Further studies are required to precisely define the effect of linagliptin on the inside-out signaling pathway leading to fibrinogen receptor activation by ADP.

Alternatively, recent studies show evidence that the pharmacological inhibition and genetic depletion of DPP-4 in animal models and human tissues is associated with antithrombogenic outcomes (Gupta et al., 2012; Krijnen et al., 2012; Cimmino et al., 2015; Cameron-Vendrig et al., 2016; Beckers et al., 2017). In most studies, the molecular mechanisms involved in DPP-4 inhibition include improved glucose uptake, increased cyclic AMP levels and activated downstream kinases, such as PKA and AKT. However, limited mechanistic data link the reduction of DPP-4 activity to improvements in platelet function independent of changes in glucose haemostasis and inflammation. Thus, our studies highlight the important roles of DPP-4 in platelet function and thrombus formation.While our study have shown the interaction between NO and DPP-4 inhibition and provided important mechanistic insights. A limitation of experiments using the carotid artery thrombosis model is that the prothrombotic effects of platelet-derived DPP-4 cannot be definitively proven to be caused by thrombin generation. Nevertheless, our current results, which demonstrated that linagliptin blocks arterial thrombosis, support the significance of our proposed molecular mechanisms in the risk of thrombosis. Another limitation is the lack of relevant data on the association between platelet and endothelium in the present study. We have not explored non-platelet mechanisms by which DPP-4 might regulate arterial thrombosis by directly affecting vascular wall cells. Recently, it has been reported that DPP-4 inhibitor anagliptin prevented FeCl3-induced arterial thrombus by rectifying an imbalance between ADAMTS13 and vWF in mice received chronic stress (Jin et al., 2020). The current *in vivo* studies did not examine plasma levels of ADAMTS13 and vWF in mice received DPP-4 inhibitor. It is acknowledged that DDP-4 inhibitor, linagliptin has been associated with arterial thrombosis through mechanisms involving ADAMTS13 and oxidative stress. Future studies are warranted to resolve these important issues.

Overall, our results broaden our understanding of the roles of DPP-4 in regulating platelet activation and thrombosis. Future studies will be required to evaluate the effects of DPP-4 inhibition and knockout approaches on platelet reactivity in DM2 and metabolic syndrome. These mechanistic studies provide new insights into therapeutic strategies to inhibit platelet abnormalities and thrombosis.

## Data Availability Statement

The raw data supporting the conclusions of this article will be made available by the authors, without undue reservation, to any qualified researcher.

## Ethics Statement

The animal study was reviewed and approved by Southwest Medical University.

## Author Contributions

All authors made substantial contributions to the conception and design of the various aspects of the prospective studies or to the acquisition, analysis or interpretation of data. All authors also contributed to drafting the article or revising it critically for important intellectual content and have given final approval of the version to be published. JW and YL are responsible for the integrity of this work as a whole, including the study design, access to data, and the decision to submit and publish the manuscript.

## Funding

This work was supported by the National Natural Science Foundation of China Grant (81172050, 81570263 to JW).

## Conflict of Interest

The authors declare that the research was conducted in the absence of any commercial or financial relationships that could be construed as a potential conflict of interest.
