# Supplementary figures and images for: Linagliptin Regulates the Mitochondrial Respiratory Reserve to Alter Platelet Activation and Arterial Thrombosis
Source: Front Pharmacol. 2020 Nov 30;11:585612. doi: 10.3389/fphar.2020.585612 (PMC7734318; doi:10.3389/fphar.2020.585612)

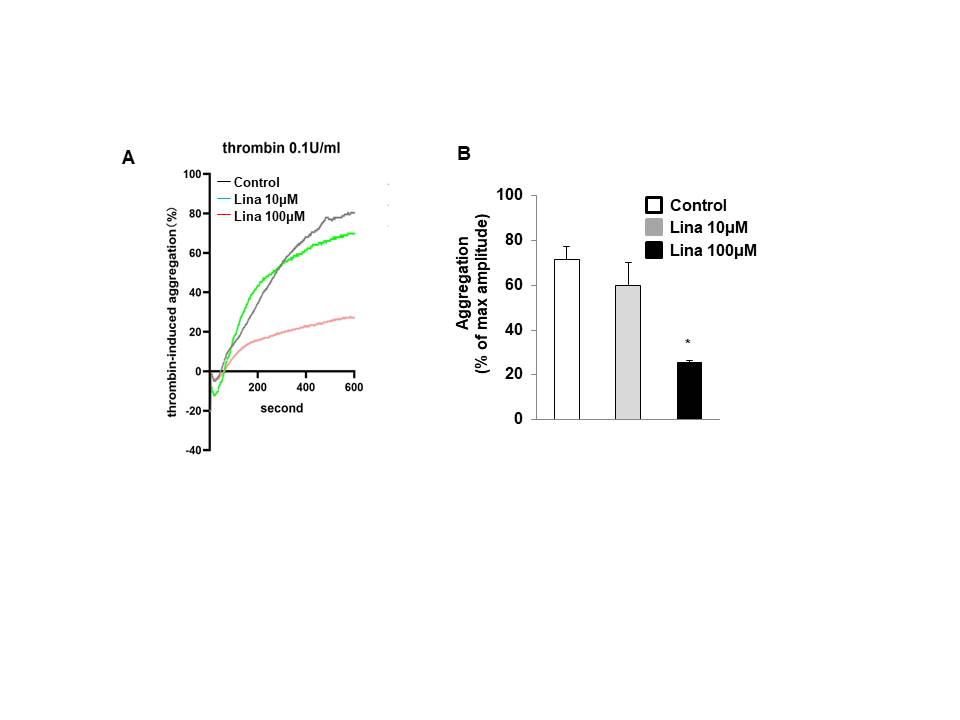

Supplement: Supplementary file 1 [file image1.jpeg]
